# Supplementary material for: A Fast, Efficient, and Tissue-Culture-Independent Genetic Transformation Method for Panax notoginseng and Lilium regale
Source: Plants (Basel). 2024 Sep 6;13(17):2509. doi: 10.3390/plants13172509 (PMC11397655; doi:10.3390/plants13172509)
Supplement: Supplementary file 1 [file plants-13-02509-s001.zip › plants-3143405-supplementary.pdf]

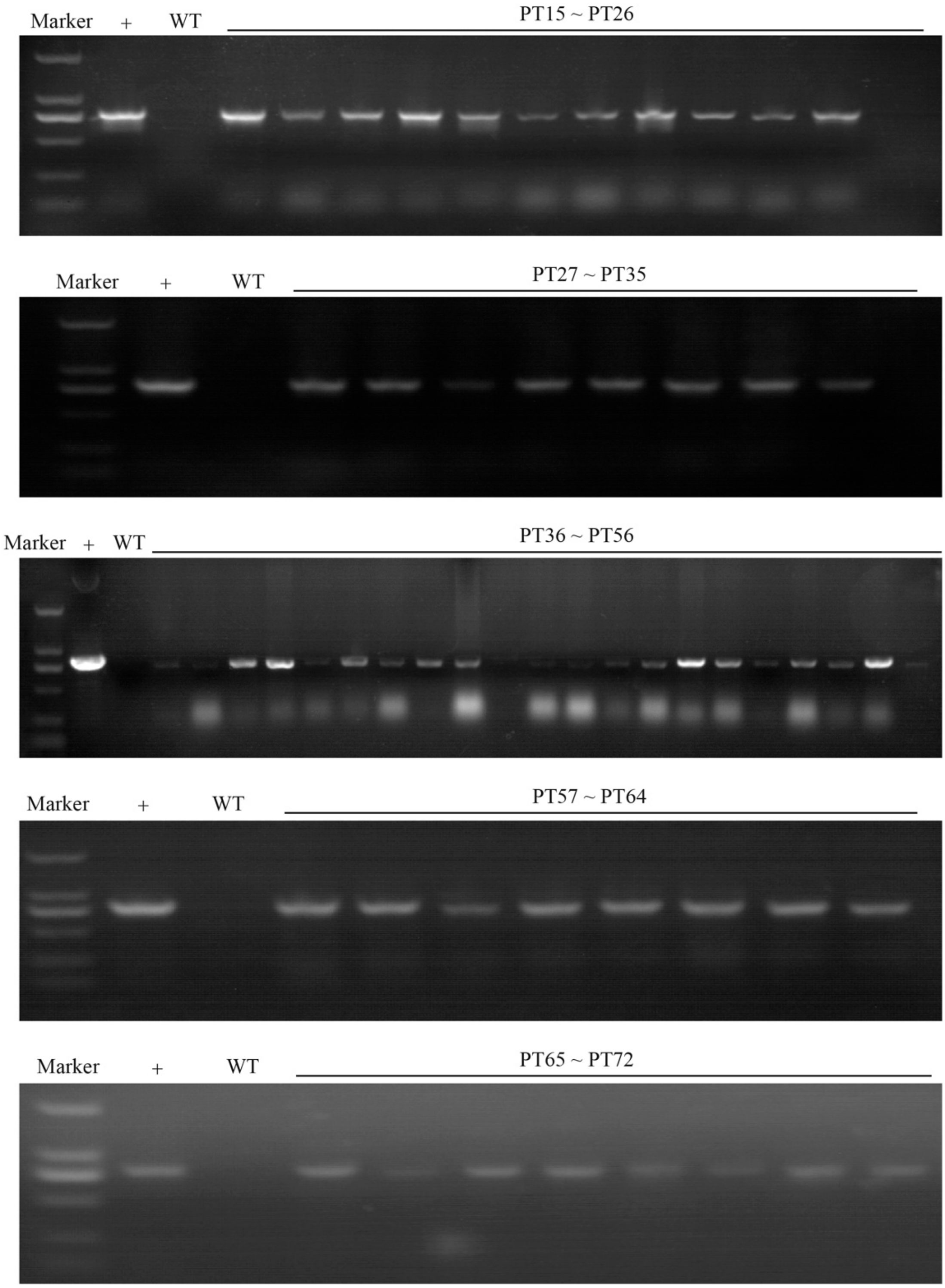

Figure S1. Gel electrophoresis results of *DsRed2* amplification based on the genomic DNA extracted from one leaf of each injected *P. notoginseng* plant. PT15-PT72, injected *P. notoginseng* plants. WT, wild type *P. notoginseng* plants. +, pCAMBIA2300::35S::DsRed2 plasmids.

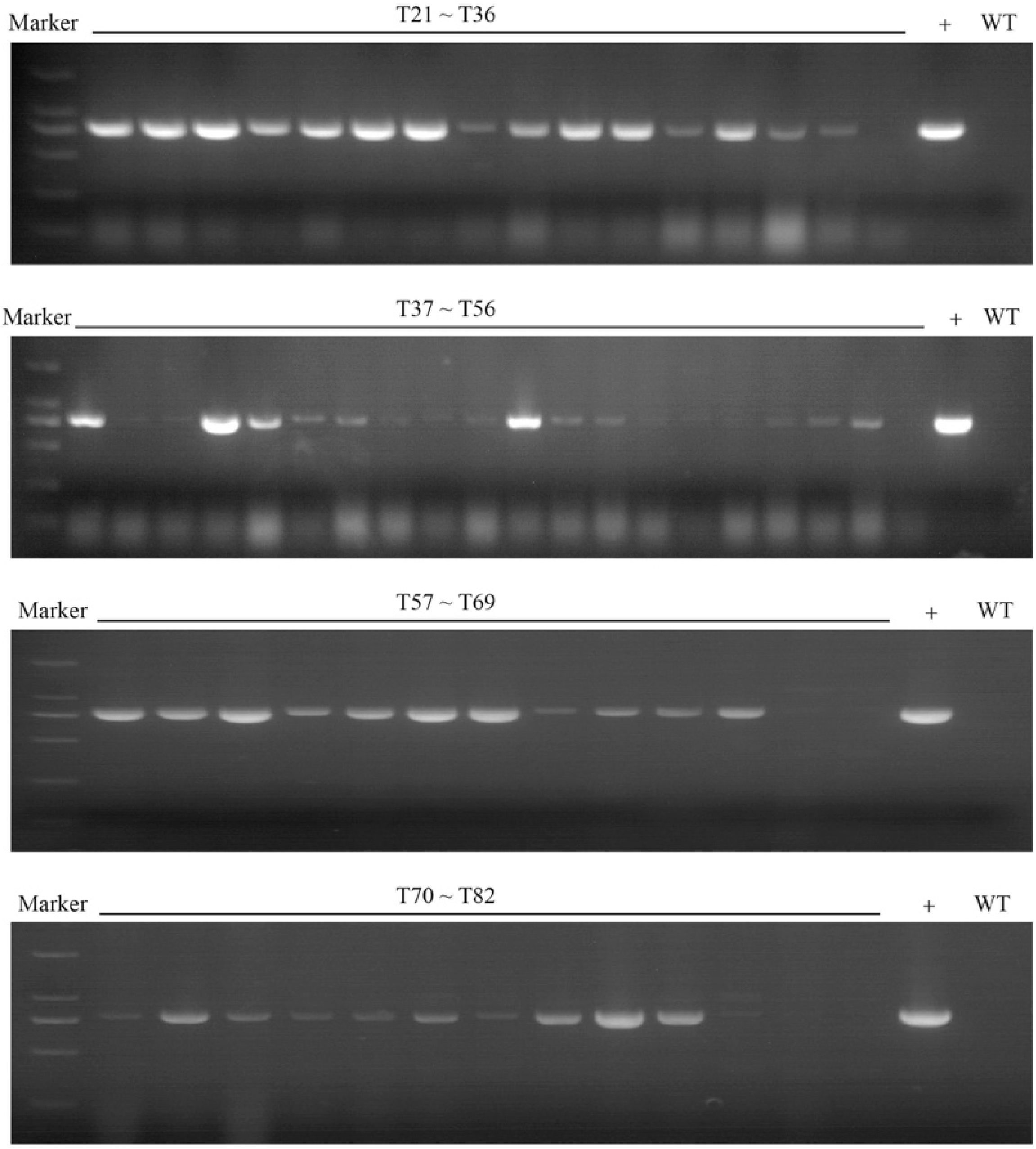

Figure S2. Gel electrophoresis showing *DsRed2* amplification from based on the genomic DNA extracted from one leaf of each injected *L. regale* plants. T-21~T-82, injected *L. regale* plants. WT, wild type *L. regale* plants. +, pCAMBIA2300::35S::DsRed2 plasmids.

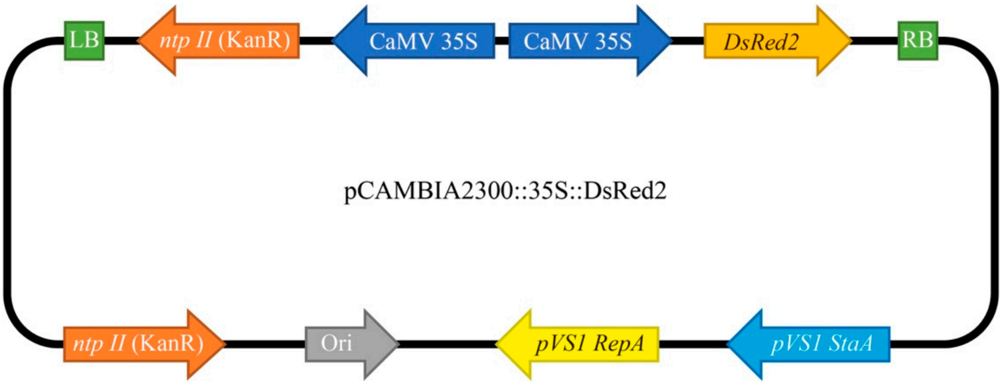

Figure S3. The schematic representation of pCAMBIA2300::35S::DsRed2 vector. LB, left border repeat from nopaline C58 T-DNA. RB, right border repeat from nopaline C58 T-DNA. *ntp II* (KanR), aminoglycoside phosphotransferase. CaMv35S, strong constitutive promoter from cauliflower mosaic virus. Ori, high-copy-number ColE1/pMB1/pBR322/pUC origin of replication. *pVS1 RepA*, replication protein from *Pseudomonas* plasmid pVS1. *pVS1 StaA*, stability protein from *Pseudomonas* plasmid pVS1.

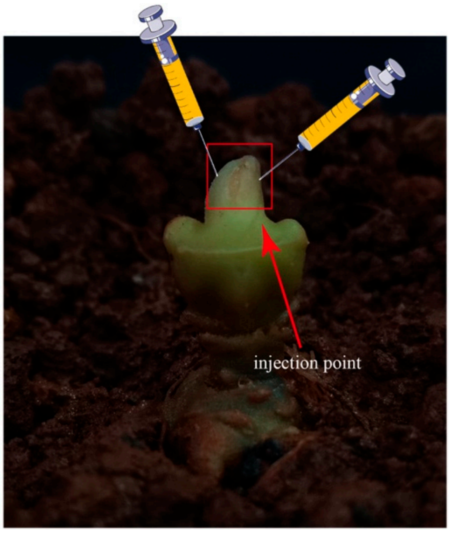

Figure S4. The injection position diagram of the *P. notoginseng* rhizome.

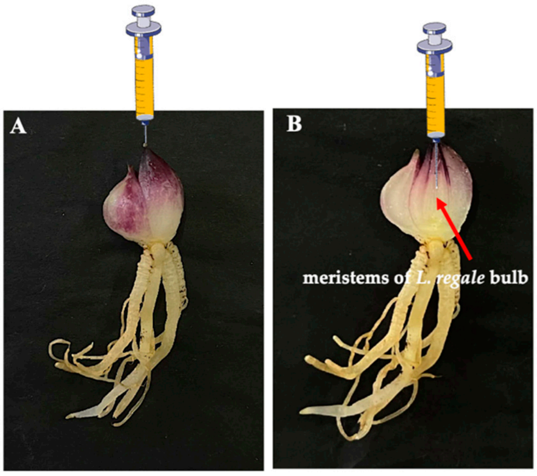

Figure S5. The injection position diagram of the *L. regale* bulb. (A) The injection position diagram of one-year old *L. regale* bulb. (B) The cross-sectional schematic diagram of injection position of *L. regale* bulb.
